# The prevalence of hyperuricemia and its correlates in Ganzi Tibetan Autonomous Prefecture, Sichuan Province, China

**DOI:** 10.1186/s12944-018-0882-6

**Published:** 2018-10-12

**Authors:** Xin Zhang, Qingtao Meng, Jiayue Feng, Hang Liao, Rufeng Shi, Di Shi, Lachu Renqian, Zeren Langtai, Yuanbin Diao, Xiaoping Chen

**Affiliations:** 1 Department of Cardiology, West China Hospital, Sichuan University, Chengdu, Sichuan 610000 People’s Republic of China; 2 Jiulong County People’s Hospital, Jiulong, 616200 Tibetan Autonomous Prefecture People’s Republic of China; 3 Ganzi Tibetan Autonomous Prefecture People’s Hospital, Kangding, 626000 Tibetan Autonomous Prefecture People’s Republic of China

**Keywords:** Hyperuricemia, Uric acid, Prevalence, Tibetan, Tibet, Risk

## Abstract

**Background:**

Hyperuricemia is a common and serious public health problem. There has been no broad epidemiological survey of hyperuricemia in China, especially in Tibetan area. This study was therefore investigated the prevalence of hyperuricemia and its correlated factors among people aged 18–85 years in Ganzi Tibetan Autonomous Prefecture, Sichuan Province, China.

**Methods:**

We carried out a cross-sectional study among 3093 participants in Ganzi Tibetan Autonomous Prefecture using questionnaires in face-to-face interviews, anthropometric measurements and biochemical tests. We included 1416 subjects with complete data including serum uric acid and medical history to analyze the prevalence of hyperuricemia and correlated factors. Hyperuricemia was defined as a fasting serum uric acid level higher than 420 μmol/L in men and 360 μmol/L in women.

**Results:**

The overall crude prevalence of hyperuricemia was 37.2%, and was greater in men than women (41% vs 34.4%, *P* = 0.011). The age-adjusted prevalence was 33.0%. Characteristics linked to hyperuricemia were farmers-herdsmen (OR: 1.749, 95% CI: 1.022–2.992), low to moderate education level (low OR:1.57, 95% CI: 1.102–2.237; moderate OR: 1.86, 95% CI: 1.167–2.963), current drinking (OR: 1.795, 95% CI: 1.193–2.702), hypertension (OR: 1.48, 95% CI: 1.091–2.006), higher body mass index (1 unit increase) (OR: 1.116, 95% CI: 1.077–1.156) and higher serum creatinine (1 unit increase) (OR: 1.046, 95% CI: 1.034–1.059). Serum uric acid was positively related to triglycerides and total cholesterol and negatively related to high density lipoprotein cholesterol in all subjects. Hyperuricemia was a risk factor for high triglyceride ((OR: 2.13, 95% CI: 1.156–3.9266) and high total cholesterol (OR: 2.313, 95% CI: 1.364–3.923) in men and for high low-density lipoprotein cholesterol (OR: 2.696, 95% CI: 1.386–5.245) in women.

**Conclusion:**

There is a high prevalence of hyperuricemia in Ganzi Tibetan Autonomous Prefecture. The government needs to prevent and manage hyperuricemia in this area.

## Background

Hyperuricemia is defined as a fasting serum uric acid higher than 420 μmol/L in men and 360 μmol/L in women [1]. It causes gout and urinary calculi and is also related to a variety of comorbidities, including cardiovascular diseases, chronic kidney disease, metabolic syndrome, diabetes mellitus [2]. Previous studies have shown that serum uric acid is affected by age, sex, ethnicity, dietary habits, environment and other factors [1].

The United States National Health and Nutrition Examination Survey 2007–2008 study showed that the prevalence of Hyperuricemia was 21.2% among men and 21.6% among women in America [3]. The China National Survey of Chronic Kidney Disease showed that the age-adjusted prevalence of hyperuricemia among Chinese adults in 2009–2010 was 8.4% (9.9% in men and 7.0% in women) [4]. A recent meta-analysis of 36 articles from 2000 to 2014 showed that the prevalence of hyperuricemia and gout in Mainland China was 13.3% [5]. Socioeconomic developments and lifestyles changes in China mean that the prevalence of hyperuricemia has increased in the last few decades, with a trend toward onset at younger ages. A previous investigation in Mainland China showed that the condition is more prevalent in men than women, in cities than rural areas, and in coastal than inland areas [1, 4]. However there have been few broad epidemiological surveys of hyperuricemia in China, especially in remote and underdeveloped areas.

Ganzi Tibetan Autonomous Prefecture is a large high altitude area of Tibet, with distinct geographic characteristics and lifestyles compared with other areas of mainland China. People tend to eat a high-salt, fat-rich diet and drink alcohol, so the cardiovascular risk factors and level of cardiovascular disease are more serious [6–8]. Most studies in this area have investigated the prevalence of cardiovascular disease and risk factors including hypertension, diabetes mellitus, overweight or obesity, and dyslipidemia in Tibetan area [6, 9, 10]. Few studies have focused on determining the prevalence and epidemiological features of hyperuricemia in this region.

It is important to obtain more epidemiological data on chronic disease and cardiovascular risk factors to improve medical and health status this area. This study therefore examined the prevalence of hyperuricemia and its correlated factors among people aged 18–85 years in Ganzi Tibetan Autonomous Prefecture, Sichuan Province, China founded by Sichuan science and technology department.

## Methods

### Study subjects

Ganzi Tibetan Autonomous Prefecture is located in western Sichuan province, China. From September 2016 to November 2017, a representative sample of participants aged 18 years and over was selected to investigate the prevalence and risk factors of chronic cardiovascular disease including hypertension, dyslipidemia, diabetes mellitus and hyperuricemia. This investigation used a multi-stage, stratified, random-cluster sampling scheme. Two counties in the prefecture (Jiulong and Seda) were selected first. In the second stage, a town was randomly selected in Jiulong county, and a town and a Buddhist college in Seda county. All the eligible participants 18 years and over were included. We excluded pregnant women, and those with malignant tumors, mental health problems or having artificial extracorporeal liver support and hemodialysis or peritoneal dialysis as a result of severe hepatic or kidney failure. In total, We included 1416 subjects with information about serum uric acid and medical history out of 3093 subjects overall. This study was approved by the Ethics committee of West China Hospital, Sichuan University (Chengdu, China). All of the participants gave informed consent after had been given information about the objectives and benefits of our study.

### Survey method and data collection

Data were collected by investigators during face-to-face interviews using a standardized questionnaire. All investigators received training in the objective of the study, the method of measurement, the significance of standardization and how to complete the questionnaire.

Blood pressure readings were taken from participants’ right arms at a time between 8:00 a.m. and 12:00 noon using a standardized automatic electronic sphygmomanometer (Omron HEM-770A). Readings were taken three times at 2-min intervals after at least 5 min of rest in a warm and quiet indoor place [11]. Systolic blood pressure (SBP) and diastolic blood pressure (DBP) were taken as the mean of three readings.

Weight and height were measured to the nearest 0.2 kg and 1 cm. Waist circumference (WC) was measured at the umbilicus using a non-elastic tape (to the nearest 0.5 cm) at the level midway between the lateral rib margin and the iliac crest in the end of a normal exhalation. Hip circumference (HC) was measured at the level of the greater trochanter [12]. Body mass index (BMI) was calculated as the weight in kilograms divided by the square root of the height in meters. Waist-to-hip ratio (WHR) was calculated as WC divided by HC.

### Laboratory measurements

Blood samples were obtained in the morning after overnight fasting. Biochemistry parameters were measured at the laboratory of West China Hospital (Chengdu, China). Total cholesterol (TC), triglycerides (TG), and fasting plasma glucose (FPG) were all determined using the enzymatic method, and high-density lipoprotein cholesterol (HDL-C) was measured using the phosphotungstic acid/magnesium chloride precipitation method. The concentration of low-density lipoprotein cholesterol (LDL-C) was calculated using the Friedewald formula. Creatinine was tested by picric acid Jaffe method and uric acid by kinetic UV assay [13].

### Definitions

Hyperuricemia was defined as a fasting serum uric acid higher than 420 μmol/L in men and 360 μmol/L in women, in line with Chinese guideline [6]. Hypertension was defined as a SBP of at least 140 mmHg, and/or a DBP of at least 90 mmHg, and/or use of antihypertensive medication. Dyslipidemia was defined using the National Cholesterol Education Program-Third Adult Treatment Panel (ATP III) criteria [14] . High TC was defined as TC of at least 6.21 mmol/L (240 mg/dL). Low HDL-C was defined as less than 1.03 mmol/L (40 mg/dL). High LDL-C was de fined as at least 4.16 mmol/L (160 mg/dL). High TG was defined as at least 2.26 mmol/L (200 mg/dL). Diabetes mellitus was diagnosed using WHO criteria: FPG of at least 7 mmol/L (126 mg/dL) and/or being treated for diabetes [15].

Current smokers were defined as smoking at least one cigarette per day during the last year [16]. Current drinkers were defined as drinking alcohol at least 12 times during the last year [17]. A vegetarian diet was defined as one that did not contain meat, poultry or fish but did contain eggs and dairy, plus plant-based foods, such as fruits, vegetables, whole grains, legumes, nuts and seeds. A non- vegetarian diet contained meat, poultry or fish, vegetables and fruits [18]. Participants were also asked whether they drank buttered tea for breakfast. People at high altitude often take only mild to moderate intensity activity because of the hypoxic environment. We classified physical activity per day by exercise time into four levels: less than 30 min as level 1, 30 min–1 h as level 2, 1–1.5 h as level 3, and more than 1.5 h as level 4. Education level was assessed as illiteracy, primary school, middle school, or high school and higher.

### Statistics

All the statistical analyses used SPSS version 23.0. Descriptive statistics were calculated for all the variables. Continuous variables were reported as mean values and standard deviations and categorical variables as numbers and percentages. The baseline data were evaluated using Student’s t test, ANOVA or the χ2 test, depending on data types. Simple correlation analysis and multiple linear regression were used to analyze the relationship between serum uric acid and the index of cardiometabolic risk factors. Multivariate logistic regression analyses were used to identify factors influencing hyperuricemia. Odds ratios (ORs) with 95% confidence intervals (CIs) were used to quantify the relationship. *P* values less than 0.05 were considered to be statistically significant.

## Results

### Baseline characteristics of study population

Table 1 shows the baseline characteristics. Current smoking and drinking were more frequent in men than women, but women were more likely to drink buttered tea and eat meat. Height, weight, heart rate, serum uric acid, glucose and serum creatinine were significantly greater for men than women. Age, serum cholesterol, serum HDL cholesterol and glucose were significantly greater in women than men, with no difference in BMI, WC, HC, WHR, SBP, DBP, serum LDL-C and serum TG between the two genders. The percentage of monks/nuns, farmers-herdsmen and ordinary residents were 37.8%, 25% and 37.2% for men and 26.3%, 41% and 32.3% for women. There were no differences in ethnic composition, education level, physical activity level and prevalence of primary hypertension.

**Table 1:** Demographic, anthropometric, and plasma biochemical characteristics of subjects in Ganzi Tibetan Autonomous Prefecture, Sichuan Province, China

Characteristics	Male	Female	Hyperuricemia	Non-hyperuricemia
	(*N* = 614)	(*N* = 802)	(*N* = 528)	(*N* = 888)
Clinic variables				
Age (years)	50.31 ± 16.84	52.21 ± 13.94 ^#^	54.98 ± 13.56	49.37 ± 15.81 ^**^
Height	165.19 ± 7.02	156.38 ± 6.72 ^##:^	160.85 ± 8.57	159.81 ± 7.82 *
Weight	69.55 ± 12.25	62.97 ± 11.39^##^	69.19 ± 12.76	63.77 ± 11.41 ^**^
BMI	25.45 ± 4.01	25.73 ± 4.29	26.69 ± 4.22	24.95 ± 4.00 ^**^
WC	85.64 ± 10.17	85.57 ± 11.90	89.28 ± 9.53	83.36 ± 11.53 ^**^
HC	94.56 ± 8.34	94.45 ± 0.08	96.17 ± 8.22	93.48 ± 8.11^**^
WHR	0.91 ± 0.84	0.91 ± 0.10	0.93 ± 0.07	0.89 ± 0.11 ^**^
SBP	134.75 ± 24.19	134.87 ± 25.51	140.06 ± 24.36	131.86 ± 24.78 ^**^
DBP	82.44 ± 15.79	81.38 ± 14.51	86.05 ± 14.51	79.48 ± 14.84 ^**^
HR	81.4 ± 14.22	79.39 ± 13.93 ^#^	79.43 ± 13.86	80.74 ± 14.20
Biochemical variables				
TC	5.03 ± 1.30	5.30 ± 1.21 ^##:^	5.58 ± 1.19	4.95 ± 1.23 ^**^
LDL -c	2.83 ± 0.92	2.89 ± 0.87	3.13 ± 0.90	2.71 ± 0.85 ^**^
HDL-c	1.29 ± 0.37	1.48 ± 0.38^##:^	1.38 ± 0.39	1.41 ± 0.38
TG	1.47 ± 1.09	1.44 ± 1.17	1.76 ± 1.27	1.28 ± 1.01 ^**^
UA	408.5 ± 105.4	333.27 ± 84.73^##:^	462.17 ± 79.88	308.50 ± 61.28 ^**^
Creatinine	84.18 ± 15.77	71.97 ± 12.71 ^##:^	83.68 ± 16.47	73.43 ± 13.25 ^**^
Glucose	5.13 ± 1.62	5.34 ± 1.37 ^#^	5.38 ± 1.49	5.18 ± 1.48 *
Lifestyle variables				
Drinking buttered tea	44%	57.8% ^##:^	64%	44.4% ^**^
Tea with salt	47.20%	61.7% ^##:^	65.10%	49.6% ^**^
Current smoking	31.10%	2.8%^##:^	22%	10.8% ^**^
Currrent drinking	20%	7.4% ^##:^	20.40%	8.2% ^**^
Eating meat	76.80%	84% ^##:^	91.50%	74.4% ^**^
Physical activity	0.067		<0.001	
Level 1	11.20%	13.60%	15.40%	10.90%
Level 2	17.80%	17%	21.90%	15.00%
Level 3	11%	15.00%	17.50%	10.90%
Level 4	59.80%	54.10%	45.20%	63.10%
Else variables				
HTN	49.20%	48.60%	63.50%	40.4% ^**^
DM	5.40%	6.20%	7.10%	5.10%
CKD	3.30%	2.50%	5.50%	0.8%*
Allopurinol	0%	0%	0%	0%
Benzbromarone	0%	0%	0%	0%
Hydrochlorothiazide	0.81%	0.99%	0.94%	0.91%
Education	0.138		0.001	
1	53.60%	59.30%	46.60%	65.10%
2	19.10%	18.30%	23.70%	15.80%
3	13.90%	11.80%	19.50%	8.70%
4	13.40%	10.60%	14.10%	10.40%
Ethnicity	0.572		<0.001	
Han	20.90%	20.80%	28.40%	16.40%
Tibetan	69.50%	71.10%	58.80%	77.10%
Yi	9.70%	8.10%	12.80%	6.50%
Occupation	<0.001		<0.001	
Monks	37.80%	26.30%	15.60%	40.60%
Farmers-herdsmen	25%	41%	39.10%	31.50%
Residents	37.20%	32.30%	45.30%	27.90%

*BMI* body mass index, *WC* waist circumference, *HC* hip circumference, *WHR* Waist-to-hip ratio, *SBP* systolic blood pressure, *DBP* diastolic blood pressure, *TC* Total cholesterol, *TG* triglycerides, *LDL-c* low-density lipoprotein cholesterol, *HDL-C* high-density lipoprotein cholesterol, *UA* uric acid, *DM* diabetes mellitus, *CKD* chronic kidney disease; Education level was assessed as illiteracy, primary school, middle school, or high school and higher for1,2,3,4; Physical activity was classified by exercise time into 4 levels, less than 30 min level 1, 30 min-1 h level 2, 1–1.5 h level 3, more than 1.5 h level 4. ^#^:*P*<0.05; ^##^:*P*<0.001, male Versus female; *:*P*<0.05; **:*P*<0.001, Hyperuricemia group Versus Non-hyperuricemia group

### Prevalence of hyperuricemia in different genders and age groups

**Fig. 1:**
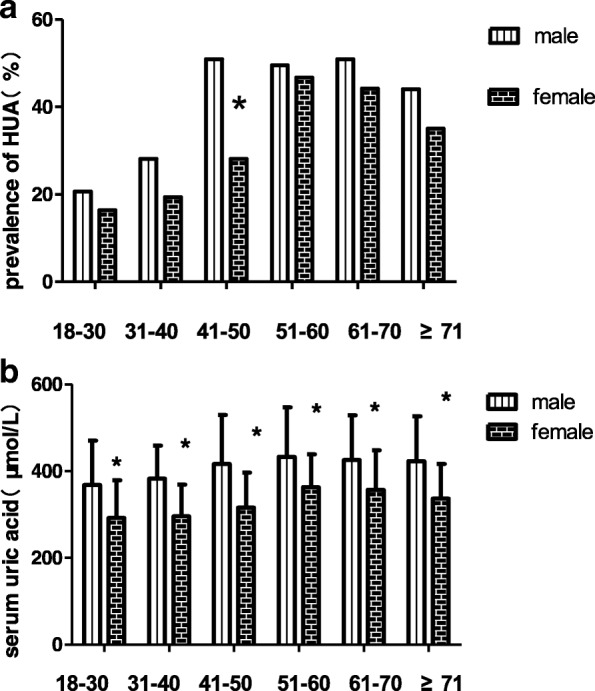
Serum uric acid level and prevalence of hyperuricemia by age and sex. a prevalence of hyperuricemia, b Serum uric acid level. #*P* < 0.05;*P < 0.001

The overall crude prevalence of hyperuricemia was 37.2%. It was more prevalent in men than women (41% vs 34.4%, *P* = 0.011), and the serum uric acid level was also higher for men than women overall (408.45 ± 105.35 vs 333.27 ± 84.73 μmol/L, *P* < 0.001) and in age cohorts. The prevalence of hyperuricemia and average serum uric acid in different age groups was shown in Fig. 1. The age-adjusted prevalence of hyperuricemia was 33.0% using the most recent population statistics for Ganzi Tibetan Autonomous Prefecture.

### Correlated factors and hyperuricemia

Multivariable logistic regression showed that being farmers-herdsmen ((OR: 1.749, 95% CI: 1.022–2.992), having low to moderate education level ((low OR: 1.57, 95% CI: 1.102–2.237; moderate OR: 1.86, 95% CI: 1.167–2.963), current drinking OR: 1.795, 95% CI: 1.193–2.702), hypertension ((OR: 1.48, 95% CI: 1.091–2.006), higher BMI (1 unit increase) (OR: 1.116, 95% CI: 1.077–1.156) and higher creatinie (1 unit increase) (OR: 1.046, 95% CI: 1.034–1.059) were related to hyperuricemia in all subjects (Table 2). For men, low education level, current drinking, higher BMI and higher serum creatinine were risk factors for hyperuricemia. Being Tibetan compared to Han and doing high levels of physical activity were protective factors for hyperuricemia in male group. For women, the multivariable logistic regression showed that being farmers-herdsmen and residents rather than nuns, moderate education level, current drinking, hypertension, higher BMI and higher serum creatinine were risk factors for hyperuricemia (Table 3).

**Table 2:** Multiple regression analyses of hyperuricemia and associated factors in all subjects

Variables	OR	95%CI
Female	0.903	(0.629–1.297)
Age(years)		
18–30	1	(reference)
31–40	0.618	(0.326–1.17)
41–50	0.818	(0.442–1.515)
51–60	0.836	(0.431–1.622)
61–70	0.893	(0.459–1.735)
≥ 71	0.601	(0.289–1.251)
Ethnicity		
Han	1	(reference)
Tibetan	0.71	(0.501–1.006)
Yi	1.054	(0.653–1.701)
Occupation		
Morks	1	(reference)
Farmers-herdsmen	1.749	(1.022–2.992)
Residents	1.697	(0.905–3.183)
Education		
1	1	(reference)
2	1.57	(1.102–2.237)
3	1.86	(1.167–2.963)
4	1.195	(0.691–2.066)
Drinking buttered tea	0.945	(0.669–1.335)
Eating meat	1.364	(0.835–2.227)
Physical activity		
Level 1	1	(reference)
Level 2	0.929	(0.589–1.464)
Level 3	0.895	(0.554–1.448)
Level 4	0.872	(0.578–1.317)
Current smoking	0.99	(0.647–1.513)
Current drinking	1.795	(1.193–2.702)
Hypertension	1.48	(1.091–2.006)
BMI	1.116	(1.077–1.156)
WHR	1.336	(0.299–5.959)
CREA	1.046	(1.034–1.059)

*BMI* body mass index, *WHR* Waist-to-hip ratio, *CREA* creatinine Education level was assessed as illiteracy, primary school, middle school, or high school and higher for1,2,3,4; Physical activity was classified by exercise time into 4 levels, less than 30 min level 1, 30 min-1 h level 2, 1–1.5 h level 3, more than 1.5 h level 4. Education level was assessed as illiteracy, primary school, middle school, or high school and higher; OR odds ratio, 95% CI: 95% confidence interval. *P < 0.05 for the independent association between hyperuricemia and each factor after adjusting for the remaining factors

**Table 3:** Multiple regression analyses of hyperuricemia and associated factors in different genders

Variables	Male	Female
	OR (95%CI)	OR (95%CI)
Age(years)		
18–30	1.00(reference)	1.00(reference)
31–40	0.673(0.297–1.521)	0.459(0.146–1.44)
41–50	1.289(0.56–2.965)	0.469(0.159–1.388)
51–60	0.721(0.277–1.878)	0.644(0.211–1.969)
61–70	0.793(0.311–2.022)	0.72(0.23–2.261)
≥ 71	0.515(0.185–1.434)	0.55(0.16–1.887)
Ethnicity		
Han	1.00(reference)	1.00(reference)
Tibetan	0.502(0.284–0.885)	0.885(0.557–1.407)
Yi	1.443(0.681–3.06)	0.929(0.47–1.838)
Occupation		
Morks	1.00(reference)	1.00(reference)
Farmers-herdsmen	1.321(0.579–3.013)	2.78(1.282–6.027)
Residents	1.1(0.404–2.994)	2.544(1.055–6.133)
Education		
1	1.00(reference)	1.00(reference)
2	1.774(1.012–3.11)	1.371(0.849–2.215)
3	1.296(0.619–2.715)	3.102(1.639–5.871)
4	1.017(0.437–2.37)	1.345(0.608–2.974)
Drinking buttered tea	1.199(0.709–2.028)	0.727(0.445–01.19)
Eating meat	1.439(0.744–2.78)	1.352(0.626–2.92)
Physical activity		
Level 1	1.00(reference)	1.00(reference)
Level 2	0.735 (0.353–1.53)	1.243(0.669–2.311)
Level 3	1.01(0.446–2.29)	0.962(0.505–1.829)
Level 4	0.468(0.24–0.911)	1.546(0.885–2.702)
Current smoking	0.809(0.483–1.357)	1.652(0.591–4.616)
Current drinking	1.1(1.014–3.14)	2.224(1.156–4.278)
Hypertension	1.33(0.822–2.154)	1.549(1.026–2.338)
BMI	1.1(1.04–1.164)	1.123(1.07–1.178)
WHR	0.833(0.054–12.76)	1.522(0.241–9.626)
CREA	1.034(1.019–1.05)	1.075(1.05–1.1)

*BMI* body mass index, *WHR* Waist-to-hip ratio, *CREA* creatinine Education level was assessed as illiteracy, primary school, middle school, or high school and higher for1,2,3,4; Physical activity was classified by exercise time into 4 levels, less than 30 min level 1, 30 min-1 h level 2, 1–1.5 h level 3, more than 1.5 h level 4. Education level was assessed as illiteracy, primary school, middle school, or high school and higher; OR: odds ratio, 95%CI: 95% confidence interval. *P < 0.05 for the independent association between hyperuricemia and each factor after adjusting for the remaining factors

### Hyperuricemia and cardiometabolic index

**Fig. 2:**
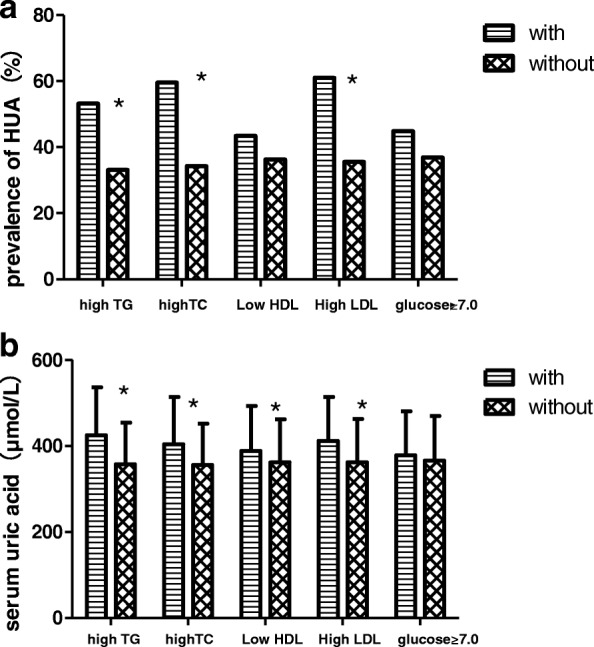
Serum uric acid level and prevalence of hyperuricemia with or without selected risk factors. **a** prevalence of hyperuricemia, **b** Serum uric acid level. #P < 0.05;*P < 0.001

Simple correlation analysis (Table 4) showed that serum uric acid was positively related to TG, TC, and LDL-c in all subjects, and for men women separately. It was negatively related to HDL-c in all subjects and women alone, but not men alone. Multiple linear regression (Table 5) showed that serum uric acid was positively related to TG and TC and negatively related to HDL-c in all subjects after adjusting for age, sex, BMI, serum creatinine, smoking, drinking alcohol, hypertension, chronic kidney disease, and diabetes mellitus. In the overall group, the prevalence of hyperuricemia was higher in subjects with high TG, high TC or high LDL-c, and the average serum uric acid was higher in subjects with high TG, high TC, high LDL-c or low HDL-c (Fig. 2). After adjusting confounding factors including age, BMI, WHR, ethnicity, occupation, education level, drinking buttered tea, eating meat, physical activity, and current smoking and drinking, the multivariable logistic regression showed that hyperuricemia was a risk factor for high TG (OR: 2.13, 95% CI: 1.156–3.926) and high TC (OR: 2.313, 95% CI: 1.364–3.923) for men and for high LDL-c (OR: 2.696, 95% CI: 1.386–5.245) for women respectively (Table 6).

**Table 4:** Cardiometabolic risk factors associated with serum uric acid levels in simple correlation

Variables	ALL		Male		Female	
	*r*	*P*	*r*	*P*	*r*	*P*
TG	0.243	<0.001	0.289	<0.001	0.23	<0.001
TC	0.247	<0.001	0.37	<0.001	0.247	<0.001
HDL-C	−0.12	<0.001	0.038	0.346	− 0.101	0.004
LDL-C	0.242	<0.001	0.331	<0.001	0.223	<0.001
glucose	0.03	0.308	0.046	0.249	0.074	0.057

*TG* triglycerides, *TC* Total cholesterol, *HDL-C*, high-density lipoprotein cholesterol, *LDL-c* low-density lipoprotein cholesterol, r: correlation coefficient; P:P value

**Table 5:** Cardiometabolic risk factors associated with serum uric acid levels in multiple linear regression

Varibles		ALL		Male		female	
		β-value	*P*	β-value	*P*	β-value	*P*
TG	Model 1	0.078	0.026	0.183	<0.001	0.07	0.149
	Model 2	0.063	0.046	0.098	0.059	0.049	0.289
TC	Model 1	0.493	<0.001	0.548	<0.001	0.472	<0.001
	Model 2	0.189	0.009	0.509	0.001	0.077	0.438
HDL-C	Model 1	−0.256	<0.001	−0.092	0.119	− 0.216	<0.001
	Model 2	−0.126	0.001	−0.115	0.5	−0.159	0.004
LDL-C	Model 1	−0.154	0.023	−0.145	0.165	−0.191	0.029
	Model 2	−0.021	0.722	−0.098	0.338	−0.006	0.937
Glucose	Model 1	−0.06	0.034	−0.097	0.019	0.006	0.878
	Model 2	−0.066	0.7	−0.083	0.49	−0.978	0.329

*TG* triglycerides, *TC* Total cholesterol, *HDL-C* high-density lipoprotein cholesterol, *LDL-c* low-density lipoprotein cholesterol; Model 1:unadjusted; Model 2: adjusted for age, sex (male = 1, female = 2), body mass index, serum creatinine, smoking (yes = 1, no = 0), drinking alcohol (yes = 1, no = 0), hypertension (yes = 1, no = 0), Chronic kidney disease (yes = 1, no = 0), diabetes mellitus (yes = 1, no = 0), P:*P* value

**Table 6:** Characteristics of subjects with and without hyperuricemia according to gender

	Male(N = 614)			Female(N = 802)		
	HAU (*N* = 250)	without HAU (*N* = 364)	OR (95%CI)	HAU (*N* = 276)	without HUA (*N* = 526)	OR (95%CI)
High TG	20.80%	8.7%^*^	2.13 (1.156–3.926)^#^	18.10%	7.2%^*^	1.549 (0.887–2.704)
High TC	32%	13.2%^*^	2.313 (1.364–3.923)^*^	27.90%	17.3%^*^	1.088 (0.699–1.694)
Low HDL	20.10%	21.80%	1.334 (0.791–2.249)	14.10%	7.3%^#^	1.706 (0.925–3.145)
High LDL	10.00%	4.50%	1.896 (0.866–4.15)	13.00%	4.4%*	2.696 (1.386–5.245)^*^
Glucose≥7.0	5.40%	5.40%	0.559 (0.217–1.439)	8.60%	4.90%	1.039 (0.463–2.333)

Adjusted for age, BMI, WHR, serum creatinine, current smoking, current drinking, ethnicity, education, physical activity, occupation, eating meat and drinking buttered tea

*BMI* body mass index, *HDL-C* high-density lipoprotein cholesterol, *LDL-C* low-density lipoprotein cholesterol, *high TC* hypercholesterolemia, *high TG* hypertriglyceridemia, *High TC* TC ≥ 6.21 mmol/L, *low HDL-C* HDL-C < 1.03 mmol/L, *high LDL-C* LDL-C ≥ 4.16 mmol/L, *High TG* TG ≥ 2.26 mmol/L.*P < 0.001; ^#^*P* < 0.05

## Discussion

This study found that the prevalence of hyperuricemia in this area of Tibet was significantly higher than other areas of Mainland China. The lifestyle in this area differs from that in inland and coastal areas of China. Firstly, in high altitude areas, food resources are limited and food diversity is insufficient when measured using the 11-item Food Diversity Score Kyoto (FDSK-11) [19]. A national survey on dietary pattern and meal behavior in China demonstrated that Tibetan ate lower legumes than other groups including Yi, Han, and Zhuang [20]. Secondly, people in Tibetan area liked eating traditional salty yak buttermilk tea, mushrooms and yak meat, which are specialties of the plateau region. Finally, drinking is a popular way to entertain guests and keeping warm fn the area, because of both the alpine climate and traditional customs. A survey of alcohol drinking behavior among native adult Tibetans in Lhasa demonstrated that the overall alcohol drinking rate was 48. 95% (64. 16% for men and 36. 00% for women), which is higher than the national average [21]. In other words, people in Tibet had a distinct dietary structure, giving priority to meat, alcohol, Fat-rich diet. Certain food, including meat, alcohol and mushrooms can increase the risks of hyperuricemia [21–23].

We found that current drinking, hypertension, higher BMI and creatinine, being farmers-herdsmen, and having low to moderate education level were risk factors for hyperuricemia. Alcohol may increase uric acid by increasing in lactic acid exchange with urate in kidneys via sodium-dependent monocarboxylate transporters SLC5A8 and SLC5A12 upregulation of urate transporter 1 (URAT1) [24]. Alcohol has also been shown to increase uric acid production by increasing adenosine triphosphate degradation to adenosine monophosphate [25]. One prospective study in Japan, confirmed that habitual alcohol intake contributed to the development of hyperuricemia, regardless of type of alcohol consumed [26]. Our study confirmed the positive association of hypertension and hyperuricemia. The prevalence of hypertension and hyperuricemia has increased worldwide in recent years. Hypertension and HUA were both related to age, metabolism and lifestyle. BMI has been used as a surrogate variable for evaluating overweight and obesity. Choi et al. confirmed that obesity was a strong risk factor for the development of hyperuricemia and gout in men [27]. A systematic review and meta-analysis of ten prospective studies confirmed that higher BMI increases the risk of gout [28]. We also found serum creatinine level was associated with hyperuricemia and the average level of serum creatinine was higher in subjects with hyperuricemia, with a higher average level of serum creatinine in those with hyperuricemia. This result was in lines with a study in the Jinan area of China [29].

Our findings also suggest that being farmers-herdsman was a risk factor for hyperuricemia in the overall group of participants. In our study, Monks/nuns had lower levels of smoking and drinking alcohol and were more likely to be vegetarian than other groups. Smoking and drinking alcohol were risk factor of hyperuricemia. The monks/nuns reported a diet of plant-based foods, such as fruits, vegetables, whole grains, legumes, nuts and seeds and eggs, and dairy. Yi-Tsen Tsai found Chinese adults in Taiwan who ate more vegetables and fruit were more likely to have a lower uric acid concentration [30]. Szeto et al. found that a long-term vegetarian diet was associated with lower concentrations of uric acid in a small group [31].

Low to moderate education level was also a risk factor of hyperuricemia in our study. Some researchers have found that well-educated individuals appeared to have a healthier and more balanced diet [32–34]. This may be because people who have low to moderate levels of education have fewer opportunities to accumulate nutritional knowledge and may, therefore, pay less attention to their dietary intake.

We also found being Tibetan rather than Han, and having a high level of physical activity were protective factors for hyperuricemia in men. A review found that the risk of developing hyperuricemia and gout varied across populations by race and ethnicity [35]. Tibetans have lived on the plateau for generations isolated from inland China and have different genetic backgrounds, with a lower level of heterozygosity, and a higher level of runs of homozygosity [36]. This could explain some at least of the ethnic differences in our study, although there is no current direct evidence of a genetic link. The relationship between physical activity and uric acid or hyperuricemia is not entirely clear. Vigorous intensity activity has been shown to increase circulating uric acid level [37]. People at high altitude, however, tend to take only mild to moderate intensity activity because of the hypoxic environment. Yamanaka et al. stated that muscle exercise, at a degree below the anaerobic threshold, did not cause major purine nucleotide degradation and may be beneficial for people with gout or hyperuricemia [38]. Another study found that moderate intensity physical activity was associated with the lower uric acid concentrations in obese individuals [39].

A lot of epidemiologic studies have reported a relationship between serum uric acid levels and a number of cardiovascular conditions, including hypertension, metabolic syndrome, coronary artery disease, cerebrovascular disease, vascular dementia, preeclampsia and kidney disease [40]. We found that serum uric acid was positively related to TG and TC and negatively related to HDL-c in all subjects, and that hyperuricemia was a risk factor for high TG and high TC in men and high LDL-c in women. Research in cell cultures and animal models have explored possible mechanisms that might explain the relationship between hyperuricemia and dyslipidemia. The first possible mechanism is inflammatory and oxidative reaction induced by uric acid in adipocytes, which was important in causing metabolic syndrome in obese mice [41].The second possible mechanism is that overexpressed xanthine oxidoreductase in adipocytes is critical to the process of adipogenesis.

### Limitations

This study has several limitations. First, it was a cross-sectional study, so could not provide any information about cause and effect. Prospective studies are required for further investigation of these findings. Second, the prevalence of hyperuricemia was assessed by a single test of blood samples, which may generate errors. Thirdly, this study was conducted only in Ganzi Tibetan Autonomous Prefecture, and so may not reflect the prevalence of hyperuricemia across the wider Tibetan area. More epidemiological studies are required to investigate the prevalence of hyperuricemia and its risk factors in high altitude areas and across Tibet. The doctors and nurses in this study were trained in how to obtain research data using a standardized measurement protocol, but measurements from a single visit might lead to incorrect values for the anthropometric indexes and language difference might result in incorrect data for medical history and lifestyle. Finally, we did not evaluate dietary structure by using a food frequency questionnaire, which is in an important component in the prevalence of hyperuricemia.

## Conclusion

In conclusion, the prevalence of hyperuricemia is relatively high in Ganzi Tibetan Autonomous Prefecture, China. Being farmers-herdsmen, having low to moderate education level, current drinking, hypertension, and having higher BMI (1 unit increase) and creatinine (1 unit increase) all contributed to hyperuricemia in this population. It is crucial for the government to pay more attention to preventing and managing hyperuricemia in this area.

## Abbreviations

BMI: Body mass index
DBP: Diastolic blood pressure
FPG: Fasting plasma glucose
HC: Hip circumference
HDL-C: High-density lipoprotein cholesterol
HUA: Hyperuricemia
LDL-C: Low-density lipoprotein cholesterol
SBP: Systolic blood pressure
TC: Total cholesterol
TG: Triglycerides
WC: Waist circumference
WHR: Waist-to-hip ratio
